# Discoidin domain receptors regulate the migration of primary human lung fibroblasts through collagen matrices

**DOI:** 10.1186/1755-1536-5-3

**Published:** 2012-02-15

**Authors:** Pedro A Ruiz, Gabor Jarai

**Affiliations:** 1 Novartis Institutes for Biomedical Research, Respiratory Disease Area, Wimblehurst Road, Horsham, RH12 5AB, UK

**Keywords:** collagen I, collagen IV, DDR, fibroblast migration, fibroblast proliferation, MMP

## Abstract

**Background:**

The two discoidin domain receptors (DDRs), DDR1 and DDR2 are receptor tyrosine kinases (RTKs) with the unique ability among RTKs to respond to collagen. We have previously shown that collagen I induces DDR1 and matrix metalloproteinase (MMP)-10 expression through DDR2 activation and a Janus kinase (JAK)2 and extracellular signal-regulated kinase (ERK)1/2-mediated mechanism in primary human lung fibroblasts suggesting that these signaling pathways play a role in fibroblast function. Fibroblasts can traverse basement membrane barriers during development, wound healing and pathological conditions such as cancer and fibrosis by activating tissue-invasive programs, the identity of which remain largely undefined. In the present work, we investigated the role of DDRs and DDR-associated signal transduction in these processes.

**Results:**

Transwell migration experiments showed that normal human lung fibroblast (NHLF) transmigration through collagen I-coated inserts is mediated by DDR2 and the DDR2-associated signaling kinases JAK2 and ERK1/2, but not DDR1. Additionally, experiments with specific small interfering (si)RNAs revealed that collagen I-induced expression of MMP-10 and MMP-2 is DDR2 but not DDR1 dependent in NHLFs. Our data showed that collagen I increases NHLF migration through collagen IV, the main component of basement membranes. Furthermore, basal and collagen I-induced NHLF migration through collagen IV-coated inserts was both DDR2 and DDR1 dependent. Finally, DDR2, but not DDR1 was shown to be involved in fibroblast proliferation.

**Conclusions:**

Our results suggest a mechanism by which the presence of collagen I in situations of excessive matrix deposition could induce fibroblast migration through basement membranes through DDR2 activation and subsequent DDR1 and MMP-2 gene expression. This work provides new insights into the role of DDRs in fibroblast function.

## Background

Discoidin domain receptors (DDRs) are non-integrin collagen receptors that belong to the receptor tyrosine kinase family [1]. There are two related DDRs, DDR1 and DDR2. DDR1 is mainly expressed in epithelial cells, particularly of the lung, kidney, mammary gland and gastrointestinal tract, whereas DDR2 is primarily found in cells of mesenchymal origin, such as fibroblasts and smooth muscle cells [1,2]. DDR1 can be activated by most collagens including collagen I to IV and VIII, while DDR2 responds to collagen I and to a lesser extent to collagen II, III and V, but does not recognize collagen IV [3]. Collagen I is the most abundant protein of interstitial connective tissue, whereas the more flexible, network-forming collagen IV is the most important structural component of basement membranes [4].

Studies with knockout mice and human carcinoma cells have shown that DDR1 and DDR2 play important roles in the expression of proinflammatory and profibrotic factors such as cyclooxygenase-2, bone morphogenetic protein (BMP)-2, BMP-5 and BMP-7, and several matrix metalloproteinases (MMPs) [3,5-8]. DDRs have been associated with processes such as extracellular matrix (ECM) remodeling, wound repair, migration, and proliferation [2,5,1,9] and studies *in vivo *and *in vitro *have implicated DDRs in various fibrotic and fibroproliferative conditions such as cancer, atherosclerosis, inflammation, arthritis, and fibrosis of the kidney, liver, skin and lung [1,2,10-16].

Fibroblast migration and proliferation with deposition of ECM proteins are main hallmarks of fibrotic and wound healing processes [17]. In order to migrate through connective tissue, fibroblasts must degrade the surrounding basement membrane and accumulating evidence suggest that fibroblast migration relies on the activity of MMPs, which remodel basement membranes by selectively degrading different components of the ECM [18].

We have previously shown that collagen I can selectively induce DDR1 expression through a DDR2-Janus kinase (JAK)2-extracellular signal-regulated kinase (ERK)1/2-mediated mechanism and independently of β1 integrins in primary normal human lung fibroblasts (NHLFs). Furthermore, our data showed that collagen I induced the expression of several profibrotic factors and matrix-degrading enzymes such as monocyte chemoattractant protein-1 (MCP-1), BMP-2, MMP-2, and MMP-10 in NHLFs and that silencing of DDR2, but not DDR1 with specific small interfering (si)RNAs inhibited collagen I-induced MMP-10 expression in these cells [19]. MMP-10 is a broad-spectrum matrix metalloproteinase that is able to degrade a wide range of components of the ECM and basement membranes including collagens III and IV, laminin, and elastin [20-22]. Similarly, MMP-2 can contribute to the breakdown of a range of ECM proteins such as fibronectin and collagens including collagen I, IV, V and × and is also responsible for the activation of other MMPs such as MMP-9, and MMP-13, playing an important role in cell migration and tissue remodeling [23]. Importantly, the involvement of DDR2 in both the expression of several MMPs and in the regulation of DDR1 expression, the only DDR isoform with the ability to recognize network-forming collagen IV, suggests an important role for DDR1 and DDR2 in tissue remodeling and disease. Based on our previous findings we hypothesized that DDR1 and DDR2 may play a role in inducing an invasive fibroblast phenotype via collagen driven mechanisms.

## Results

### Collagen I induces migration of primary human lung fibroblasts through collagen IV

**Figure 1:**
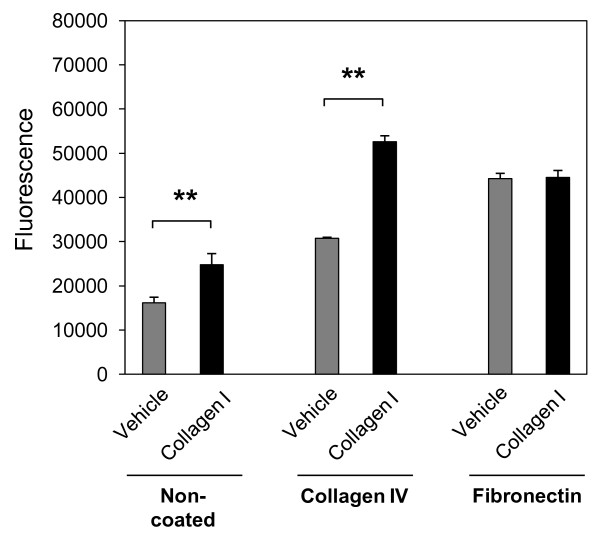
**Collagen I induces normal human lung fibroblast (NHLF) migration through collagen IV**. NHLFs were grown on non-coated 8.0 μm polycarbonate inserts, or inserts coated with collagen IV (10 μg/cm^2^) or fibronectin (5 μg/cm^2^). Cells were serum-starved for 24 h and incubated with collagen I (25 μg/mL) or vehicle (acetic acid, 0.1 M) for 24 h. The bottom chamber was treated with cell dissociation buffer and acetomethoxycalcein (calcein AM) for 1 h at 37°C. Fluorescence of solution with detached cells was measured at 485 nm excitation and 520 nm emission. Results are representative of mean fold increase ± SD of two independent experiments performed in triplicate (n = 6, **P < 0.01).

There is a large body of evidence highlighting the impact of various ECM proteins on cellular function including cell differentiation, attachment, and migration. To address the question whether collagen I is able to induce NHLF migration we performed transwell migration assays using inserts coated with different ECM proteins including network-forming collagen IV and fibronectin, as well as non-coated inserts. Our results show that the level of constitutive NHLF migration was higher through inserts coated with fibronectin compared to non-coated, and collagen IV-coated inserts, and was not further enhanced upon collagen I stimulation. In contrast, collagen I significantly enhanced constitutive NHLF transmigration through both non-coated and collagen IV-coated inserts (Figure 1). These results indicate that different ECM components have different effects on fibroblast migration and suggest a possible link between collagen I-induced gene expression and fibroblast migration through basement membranes.

### Collagen I-induced MMP-2 expression is DDR2 but not DDR1 dependent in NHLFs

**Figure 2:**
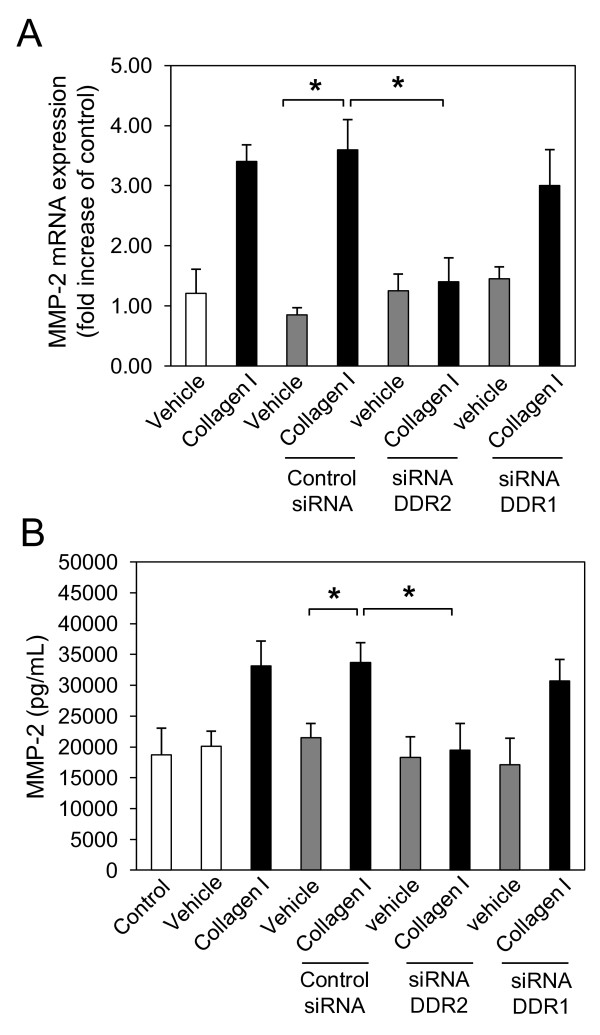
**Collagen I induces matrix metalloproteinase (MMP)-2 expression through discoidin domain receptor (DDR)2, but not DDR1 in normal human lung fibroblasts (NHLFs)**. NHLFs were reverse transfected with negative control small interfering (si)RNA, and DDR2-specific and DDR1-specific siRNA using lipofectamine RNAiMAX. At 48 h after transfection, NHLFs were serum-starved for 24 h and incubated with collagen I (25 μg/mL) or vehicle (acetic acid, 0.1 M) for 16 h. Total RNA was isolated, reverse transcribed and real-time quantitative PCR was performed using the TaqMan system with specific primers and TaqMan probes for human, MMP-2 **(A)**, and glyceraldehyde 3-phosphate dehydrogenase (GAPDH). The expression changes (fold increase) were calculated relative to unstimulated control cells after normalizing with GAPDH. Results are representative of mean fold increase ± SD of three independent experiments performed in triplicate (n = 9, *P < 0.05). NHLFs were transfected with DDR2-specific or DDR1-specific siRNAs or negative control siRNA prior to starvation and 16 h of collagen I stimulation. MMP-2 **(B) **protein was measured in the culture supernatant by ELISA. Results are representative of mean fold increase ± SD of three independent experiments performed in triplicate (n = 9, *P < 0.05).

DDRs have been implicated in the expression of several factors involved in cell migration and wound healing such as MMPs [24]. We have previously shown that collagen I induces MMP-10, and MMP-2, but not MMP-9 mRNA expression in NHLFs. Furthermore, collagen I-induced MMP-10 expression was shown to be DDR2 but not DDR1 dependent in these cells [20]. In the present study, collagen I-induced MMP-2 mRNA (Figure 2A) and protein expression (Figure 2B) in NHLFs was significantly inhibited by DDR2-specific, but not DDR1-specific, siRNAs. Taken together, these results suggest an important role of DDR2 in collagen I-induced expression of both MMP-10 and MMP-2.

### Constitutive and collagen I-induced NHLF migration through collagen IV is DDR2 dependent

**Figure 3:**
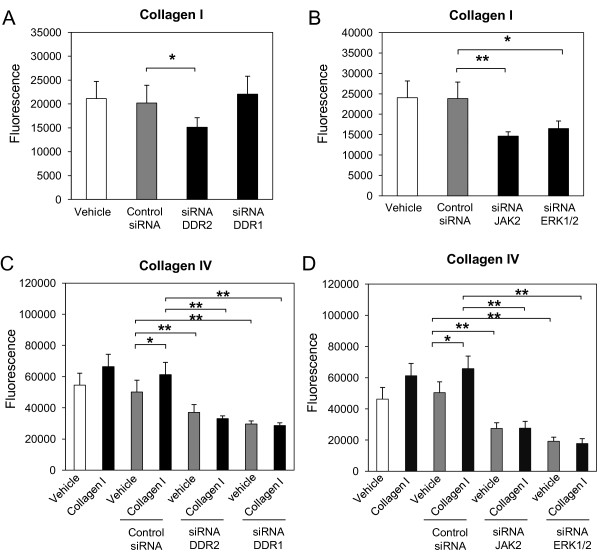
**Normal human lung fibroblast (NHLF) migration through collagen I and collagen IV is discoidin domain receptor (DDR)2-dependent**. NHLFs were reverse transfected with negative control small interfering (si)RNA, or specific siRNA for DDR2, DDR1 **(A, C)**, Janus kinase (JAK)2, or extracellular signal-regulated kinase (ERK)1/2 **(B, D) **using lipofectamine RNAiMAX. At 48 h after transfection, NHLFs were serum-starved for 24 h and transferred to 8.0 μm polycarbonate inserts coated with collagen I (10 μg/cm^2^) (A, B) or collagen IV (10 μg/cm^2^) (C, D). Where indicated, cells were incubated with collagen I (25 μg/mL) or vehicle (acetic acid, 0.1 M) for an additional 16 h **(E)**. The bottom chamber was treated with cell dissociation buffer and acetomethoxycalcein (calcein AM) for 1 h at 37°C. The fluorescence of solution with detached cells was measured at 485 nm excitation and 520 nm emission. Results are representative of mean fold increase ± SD of two independent experiments performed in triplicate (n = 6, *P < 0.05, **P < 0.01).

Since DDR2-induced DDR1 is involved in collagen IV recognition, and both MMP-10 and MMP-2 are involved in collagen IV degradation, we investigated whether collagen I-induced activation of DDR2 and downstream signaling play a role in fibroblast transmigration through collagen IV. We explored the role of DDR2 and 1, as well as the DDR2-associated kinases JAK2, and ERK1/2 in NHLF migration through collagen I and collagen IV matrices using transwell migration experiments. As shown in Figure 3A, NHLF migration through collagen I-coated inserts was significantly inhibited in the presence of DDR2-specific, but not DDR1-specific, siRNA, suggesting a role for DDR2 in the modulation of fibroblast migration though collagen I. While both DDR1 and DDR2 have been shown to recognize collagen I [1,25] our results suggest that only DDR2 is necessary for maximum migration of NHLFs through collagen I matrices. Accordingly, migration through collagen I-coated inserts was also significantly inhibited in NHLFs transfected with JAK2-specific and ERK1/2-specific siRNA (Figure 3B) further confirming the role of DDR2 signaling in substrate recognition and subsequent migration of fibroblasts through collagen I-containing matrices. Furthermore, basal as well as collagen I induced migration of NHLFs through collagen IV was significantly inhibited by both DDR2-specific and DDR1-specific siRNAs (Figure 3C), reflecting the unique ability of DDR1 to recognize collagen IV. Interestingly, and importantly with regard to the functional role of DDR2 in fibroblast migration, activation of DDR2 and the DDR2-associated kinases JAK2, and ERK1/2 (Figure 3D) appear to be involved in fibroblast migration through collagen IV even though DDR2 is not able to recognize collagen IV.

### MMP-2 is involved in collagen I-induced NHLF migration through collagen IV

**Figure 4:**
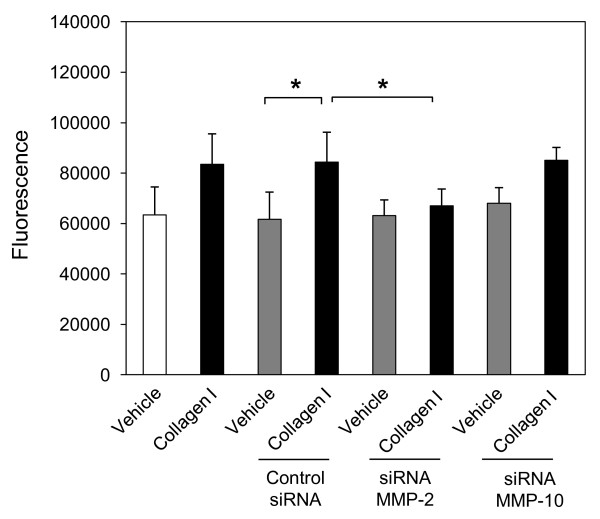
**Normal human lung fibroblast (NHLF) migration through collagen IV is matrix metalloproteinase (MMP)-2-dependent**. NHLFs were reverse transfected with negative control small interfering (si)RNA, or specific siRNA for MMP-10, or MMP-2 using lipofectamine RNAiMAX. At 48 h after transfection, NHLFs were serum-starved for 24 h and transferred to 8.0 μm polycarbonate inserts coated collagen IV (10 μg/cm^2^). Cells were incubated with collagen I (25 μg/mL) or vehicle (acetic acid, 0.1 M) for an additional 16 h. The bottom chamber was treated with cell dissociation buffer and acetomethoxycalcein (calcein AM) for 1 h at 37°C. The fluorescence of solution with detached cells was measured at 485 nm excitation and 520 nm emission. Results are representative of mean fold increase ± SD of two independent experiments performed in triplicate (n = 6, *P < 0.05).

Since collagen I-induced expression of both MMP-10 and MMP-2 is DDR2 dependent, and DDR2 appears to be involved in NHLF transmigration through collagen IV, we investigated whether collagen I-induced NHLF migration is also dependent on MMP-10 or MMP-2. As shown in Figure 4, NHLF migration was significantly inhibited in the presence of MMP-2-specific, but not MMP-10-specific, siRNA, indicating a crucial role of collagen I/DDR2-induced MMP-2 expression in NHLF migration through collagen IV.

### NHLF proliferation is DDR2, but not DDR1 dependent

**Figure 5:**
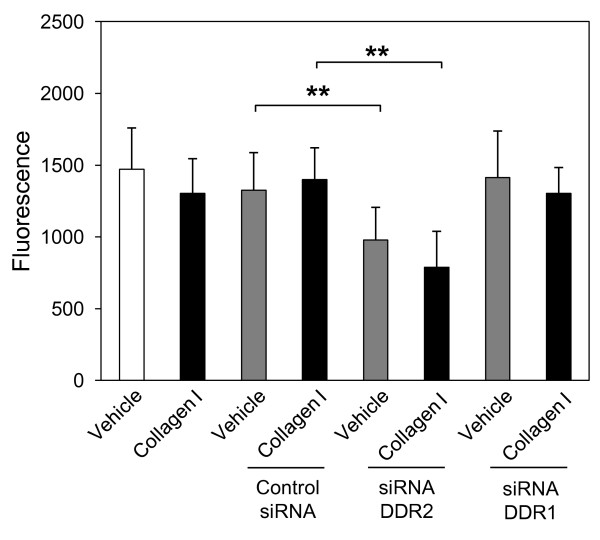
**Normal human lung fibroblast (NHLF) proliferation is discoidin domain receptor (DDR)2, but not DDR1 dependent**. NHLFs were reverse transfected with DDR2-, DDR1-specific small interfering (si)RNA or negative control siRNA using lipofectamine RNAiMAX. At 48 h after transfection, NHLFs where transferred to a 96-well black walled plate at 3,000 cells/well. Cells were serum-starved for 24 h and incubated with collagen I (25 μg/mL) or vehicle (acetic acid, 0.1 M) for an additional 16 h. Cell proliferation was determined using the DELFIA proliferation assay kit and measuring time-resolved fluorescence at 340 nm excitation and 615 nm emission. Results are representative of mean fold increase ± SD of two independent experiments performed in triplicate (n = 6, **P < 0.01).

In addition to enhanced migratory capacity, increased proliferation can also contribute to a more invasive fibroblast phenotype. We next investigated if collagen stimulation increases fibroblast proliferation and the role of DDR1 and DDR2 in this process. As shown in Figure 5 collagen I failed to induce NHLF proliferation, suggesting that collagen I-induced transmigration of NHLFs through collagen IV is not due to enhanced cell proliferation but to an increased migratory capacity of these cells. However, DDR2 appears to play a role in collagen-independent fibroblast proliferation as transfection of NHLFs with DDR2-specific, but not DDR1-specific, siRNA significantly inhibited constitutive NHLF proliferation (Figure 5). These data suggest an important role for DDR2 in fibroblast proliferation, and also highlights different roles for DDR1 and DDR2 in NHLF function.

## Discussion

In this study we show that constitutive and collagen I-induced NHLF migration through collagen IV is mediated by both DDR2 and DDR1. Additionally, silencing of the DDR2-associated kinases JAK2 and ERK1/2 with specific siRNAs inhibited migration of NHLFs through both collagen I-containing and collagen IV-containing matrices, strengthening the hypothesis that DDR2 signaling is important for human fibroblasts to enable them to recognize and degrade collagen IV. Furthermore, we have also shown that only DDR2 appears to be required for NHLF migration through collagen I.

We have previously shown that collagen I induces DDR1 and MMP-10 expression through the activation of DDR2 in primary human lung fibroblasts suggesting a key role for both DDR1 and DDR2 in fibroblast function in situations associated with excessive matrix deposition, such as fibrosis and wound healing. Knockdown experiments using specific siRNAs also confirmed an important role for both JAK2 and ERK1/2 in both constitutive and collagen I-induced DDR1 expression. Importantly, basal protein expression levels of DDR1 as well as constitutive phosphorylation of JAK2 and ERK1/2 were reduced in NHLFs in the presence of DDR2-specific siRNA, suggesting a link between DDR2 activation, JAK2 and ERK1/2 phosphorylation, and DDR1 expression [20].

The interaction of fibroblasts with the ECM and their subsequent migration into regions of injury and remodeling are major factors that contribute to wound healing and fibrosis [26]. DDRs have been shown to play an important role in cell adhesion, migration, proliferation and ECM remodeling by controlling the expression and activity of MMPs [1,27]. For example, Olaso *et al. *have demonstrated that skin fibroblasts from DDR2 knockout mice present impaired proliferation and migration through a reconstituted basement membrane concomitantly with the expression of MMP-2 [7]. Nevertheless, the functional significance of DDR activation in human lung fibroblasts has not been extensively characterized.

Together with MMP-10, type IV collagenases such as MMP-2 and MMP-9 are also able to degrade collagen IV and have been implicated in cell migration [28]. We have previously shown that collagen I induces the expression of MMP-10 and MMP-2, but not MMP-9 in primary human lung fibroblasts. Furthermore, our finding that DDR2-mediated collagen I induction of MMP-10 expression was also JAK2 and ERK1/2 dependent in NHLFs highlights the importance of these pathways in collagen I-induced expression of ECM degrading metalloproteinases [19]. MMP-10 has been shown to play a major role in tissue remodeling as it is not only able to degrade collagen III and IV, gelatin, proteoglycans and elastin [21,22], but is also responsible for the activation of other MMPs such as MMP-1, MMP-8, and MMP-9 [29]. In the present study we have shown that, together with MMP-10, collagen I-induced MMP-2 mRNA and protein expression are DDR2 but not DDR1 dependent. MMP-2 is not only able to degrade a wide range of ECM components such as elastin, fibronectin and most collagens but is also involved in the processing of growth factors and cytokines such as tumor necrosis factor and interleukin-1β, into their biologically active forms [30,31]. Experiments with MMP-10-specific and MMP-2-specific siRNA showed that collagen I-induced NHLF transmigration through collagen IV-coated inserts is MMP-2 but not MMP-10 dependent in NHLFs. MMP-2 has been shown to function as an autocrine regulator of proliferation and migration in human keratinocytes [28], and human airway smooth muscle cells [32]. Several studies suggest that while matrices rich in fibrillar collagens maintain fibroblasts in an active state, basement membrane proteins slow collagen production and receptor expression [33,34]. Thus, degradation of the basement membrane by fibroblast-derived MMPs such as MMP-2 is likely to induce continued interstitial collagen production in fibroblasts.

The finding that the expression of DDR1 and MMP-2 in NHLFs is DDR2 dependent led us to hypothesize that constitutive and collagen I-induced activation of DDR2 could increase the ability of NHLFs to recognize and degrade basement membrane collagen IV, thereby facilitating fibroblast migration through connective tissue. While we cannot formally rule out the possibility that in our experimental system collagen I coating impacts cellular adhesion and thereby modifies migration in a DDR-independent manner, our data strongly suggest a DDR2-mediated mechanism.

DDR2 has been reported to be only activated by fibril-forming collagens and collagen × however DDR1 can recognize a wide range of collagen types, including both fibril-forming collagen I and network-forming collagen IV, the main component of basal lamina [26,35]. DDR2 is much more abundantly expressed in fibroblasts than DDR1 and constitutive expression of DDR1 in NHLFs where DDR2 has been silenced does not seem to be sufficient for NHLFs to migrate through collagen I. Furthermore, our results on NHLFs transfected with DDR1-specific siRNA suggest that primarily DDR2 is responsible for recognizing collagen I in NHLFs, further strengthening the critical role of DDR2 in fibroblast migration. In contrast, constitutive and collagen I-induced DDR1 expression seems to be involved in fibroblast migration through collagen IV confirming previous reports showing a key role of DDR1 in fibroblast migration [36]. Interestingly, the silencing of DDR2, which does not recognize collagen IV [25], and the DDR2-associated kinases JAK2, and ERK1/2 also abrogated constitutive and collagen I-induced migration of NHLFs through collagen IV suggesting that the role of DDR2 in NHLF migration through collagen IV-coated inserts is likely to be due to constitutive and collagen I-induced DDR2 activation, and DDR2-dependent signal transduction and gene expression. Interestingly, DDR2-specific, but not DDR1-specific, siRNA also inhibited NHLF proliferation suggesting a wider role for DDR2 in fibroblasts function.

## Conclusions

In the present work we show that, while DDR1 is involved in NHLF migration through collagen IV, DDR2 modulates NHLF migration through both fibril-forming collagen I and network-forming collagen IV matrices. Our results suggest that collagen I-induced DDR2 activation and subsequent DDR1 and MMP-2 expression further equips human lung fibroblasts with the necessary machinery to recognize and degrade collagen IV, thereby facilitating fibroblast transmigration through basement membranes. Furthermore, our data show that the proliferation of NHLFs is also DDR2 dependent, but not DDR1 dependent, suggesting specific roles for DDR1 and DDR2 in human lung fibroblast function.

## Methods

### Cell culture and reagents

NHLFs (Lonza, Basel, Switzerland) were grown in Dulbecco's modified Eagle medium (DMEM) supplemented with 15% fetal bovine serum (FBS) and 1% penicillin/streptavidin (Life Technologies, Paisley, UK) in a humidified 5% CO2 atmosphere at 37°C. Prior to treatment, cells were serum starved overnight. Cells were stimulated with collagen I from rat tail (25 μg/mL) or vehicle (acetic acid, 0.1 M).

### RNA isolation and real-time quantitative reverse transcription (qRT)-PCR

RNA from NHLFs was extracted using RNeasy Plus Kit (Qiagen, Crawley, UK) according to manufacturer's instructions. For reverse transcription, 100 ng of total RNA was added to 50 μL of reaction buffer containing RT-PCR Buffer, MgCl_2 _(5.5 mM), desoxyribonucleoside triphosphate mixture (500 μM), random hexamers (2.5 μM), RNase inhibitors (0.4 U/μL) and MultiScribe Reverse Transcriptase (1.25 U/μL) (all reagents from Applied Biosystems), and incubated for 10 minutes at 25°C followed by 30 minutes at 48°C and 5 minutes at 95°C. Real-time PCR was performed using TaqMan system 7900HT (Life Technologies, Paisley, UK). TaqMan probes and primers used for the reaction is as follows, DDR1: 5'-GCGTCTGTCTGCGGGTAGAG-3', (forward) 5'-ACGGCCTCAGATAAATACATTGTCT-3' (reverse), 6-FAM-AGGGATGGACTCCTGTC-MGB (TaqMan probe) (99 bp), DDR2: 5'-TGTTCCTGCTGCTGCCTATCTT-3' (forward), 5'-AGGATAGCGGCATATAGCTGGAT-3' (reverse), 6-FAM-AGTTCTGCAAAAGCTCAGGT-MGB (TaqMan Probe) (68 bp), MMP-10: 5'-TCACAGAGCTCGCCCAGTT-3' (forward), 5'-CGTAGAGAGACTGAATGCCATTCA-3' (reverse), 6-FAM-CCTTTCGCAAGATGAT-MGB (TaqMan Probe) (63 bp), MMP-2: 5'-CGTCTGTCCCAGGATGACATC-3' (forward), 5'-TGTCAGGAGAGGCCCCATAG-3' (reverse), 6-FAM-AGGGCATTCAGGAGC-MGB (TaqMan Probe) (58 bp). For real-time qRT-PCR 1 μL cDNA was added to a total volume of 20 μL of reaction buffer containing TaqMan Fast Universal master mix (Life Technologies, Paisley, UK), TaqMan probes (0.3 μM) and forward and reverse primers (0.9 μM). PCR was performed by denaturation at 95°C for 20 s, followed by 40 cycles of 95°C for 1 s and 60°C for 20 s. The expression changes (fold increase) were calculated relative to unstimulated control cells using the crossing points of the log linear portion of the amplification curve after normalizing with glyceraldehyde 3-phosphate dehydrogenase (GAPDH) endogenous control (VIC/MGB) (Life Technologies, Paisley, UK).

### Small interference RNA (siRNA) and transfection

Negative control 2# siRNA, human DDR2-specific siRNA, human DDR1-specific siRNA, human MMP-10-specific siRNA, and human MMP-2-specific siRNA were synthesized by Applied Biosystems. The sequence of DDR2 siRNA is as follows: 5'-GCACUGUCAGUUACACCAATT-3' (sense) and 5'-UUGGUGUAACUGACAGUGCGT-3' (antisense). The sequence of DDR1 siRNA is as follows: 5'-GGCUAUGCAGGUCCACUGUTT-3' (sense) and 5'-ACAGUGGACCUGCAUAGCCTG-3' (antisense). The sequence of MMP-10 siRNA is as follows: 5'-GAGAAUAUCUGUUCUUUAATT-3' (sense) and 5'-UUAAAGAACAGAUAUUCUCCC-3' (antisense). The sequence of MMP-2 siRNA is as follows: 5'-GGAAAAGAUUGAUGCGGUATT-3' (sense) and 5'-UACCGCAUCAAUCUUUUCCGG-3' (antisense). JAK2-specific and ERK1/2-specific siRNAs were obtained from Cell Signaling Technologies. For siRNA delivery, NHLFs were reverse transfected with 10 nM siRNA using lipofectamine RNAiMAX (Life Technologies, Paisley, UK) according to manufacturer's instructions. At 48 h after transfection, cells were serum starved for 24 h prior to treatment with collagen I (25 μg/mL) or vehicle (acetic acid, 0.1 M). Transfection of NHLFs with specific siRNAs suppressed DDR2 by 91.7% (*P *= 0.002), DDR1 by 85.7% (*P *= 0.008), JAK2 by 93.5% (*P *= 0.003), ERK1/2 by 90% (*P *= 0.004), MMP-10 by 88.2% (*P *= 0.006), and MMP-2 by 90.7% (*P *= 0.004), versus control.

### Western blotting and immunoprecipitation

NHLFs were lysed in radioimmune precipitation assay buffer containing 50 mM Tris (pH 8), 150 mM NaCl, 1% Triton X-100, 0.5% deoxycholate acid, 0.1% SDS, proteinase inhibitors (Roche Diagnostics, Burgess Hill, UK) and protease inhibitors (Roche Diagnostics, Burgess Hill, UK). Cell debris was removed by centrifugation and supernatants were denatured at 94°C in 4 × NuPAGE sample buffer (Life Technologies, Paisley, UK) containing 5% 2-mercaptoethanol. Protein samples (20 μg) were subjected to electrophoresis on a 4-10% Bis-Tris (MOPS) NuPAGE gel (Life Technologies, Paisley, UK) and blotted onto a nitrocellulose membrane (Life Technologies, Paisley, UK). Membranes were blocked for 1 h in Tris-buffered saline (TBS), 0.1% (v/v) Tween-20 and 5% (w/v) non-fat dried milk. Immunodetection was carried out using anti-phospho-ERK1/2 (T185+Y187) (Life Technologies, Paisley, UK), anti-ERK1/2 (Cell Signaling Technologies, Danvers, MA, USA), anti-phospho-JAK2 (Y1007/Y1008) (Abcam, Cambridge, UK), anti-JAK2 (Santa Cruz Heidelberg, Germany,), anti-DDR1, anti-DDR2 (both from R&D Systems, Abingdon, UK), and anti-GAPDH (Santa Cruz, Heidelberg, Germany). The bands were visualized using the ECL system (GE Healthcare Life Sciences, Little Chalfont, UK).

### Enzyme-linked immunosorbent assay (ELISA) analysis

The concentration of pro-MMP-2 and mature MMP-2, and pro-MMP-10 in NHLFs culture supernatants was determined by MMP-2, and MMP-10 Quantikine ELISA (R&D Systems), respectively, according to manufacturer's instructions.

### Cell migration assay

NHLFs were seeded into polycarbonate inserts with an 8.0 μm pore size in a 24-well plate (Corning, Corning, NY, USA) at 75,000 cells/well. Where indicated, inserts were coated with collagen I (10 μg/cm^2^), collagen IV (10 μg/cm^2^) (both from Sigma-Aldrich, Gillingham, UK) or human plasma-derived fibronectin (5 μg/cm^2^) (R&D Systems, Abingdon, UK). Cells were serum starved overnight, and where indicated collagen I (25 μg/mL) was added to top and bottom chamber for 16 h. Bottom chamber was treated with cell dissociation buffer (Life Technologies, Paisley, UK) and acetomethoxycalcein (calcein AM; Trevigen, AMS Biotechnology, Abingdon, UK) for 1 h at 37°C. The suspension of detached cells was transferred to a 96-well plate and fluorescence was measured at 485 nm excitation and 520 nm emission.

### Cell proliferation assay

Cell proliferation was determined using the DELFIA proliferation assay kit (PerkinElmer, Cambridge, UK) according to manufacturer's instructions. Briefly, NHLFs were seeded into a 96-well black walled plate at 3,000 cells/well and grown in humidified 5% CO2 atmosphere at 37°C. Cells were serum starved overnight, and incubated with collagen I (25 μg/mL) or vehicle (acetic acid, 0.1 M) for 16 h. NHLFs were incubated with 5-bromo-2'-deoxyuridine (BrdU) for an additional 16 h, fixed and incubated with anti-BdrU labeled with europium for 90 minutes. Time-resolved fluorescence was measured at 340 nm excitation and 615 nm emission.

### Statistical analysis

Data were expressed as the mean ± SD of six or more independent experiments. Statistical analysis was performed using one-way analysis of variance (ANOVA) followed by the Tukey test. Significance was set as *P *< 0.05.

## Competing interests

The authors declare that they have no competing interests.

## Authors' contributions

PAR carried out the experiments and performed the data analysis. GJ and PAR designed the experiments, interpreted the data and wrote the final manuscript. Both authors read and approved the final manuscript.
